# Interoception Within the Context of Impulsivity and Addiction

**DOI:** 10.1007/s40429-023-00482-7

**Published:** 2023-04-29

**Authors:** Aleksandra M. Herman

**Affiliations:** Laboratory of Brain Imaging, Nencki Institute of Experimental Biology of the Polish Academy of Sciences, Pasteur 3 St, Warsaw, Poland

**Keywords:** Interoception, Addiction, Impulsivity, Self-control, Homeostasis, Afferent signals

## Abstract

**Purpose of Review:**

The goal of this review is to examine the relationship between impulsivity and interoception in addiction, to summarize the current understanding of the topic, identify any gaps in knowledge, and provide directions for future research.

**Research Findings:**

Interoception may be a contributing factor to impulsive behaviour and, thus, addiction. Substance abuse can negatively impact the brain’s ability to process interoceptive information and impact the reward system, leading to decreased sensitivity to natural rewards and increased sensitivity to drugs. There is potential for new therapies, such as mindfulness, interoceptive training, brain stimulation, or vagal nerve stimulation to target both impulsivity and interoception in the treatment of addiction.

**Summary:**

Despite a growing interest in interoception in addiction research, further research is needed to better understand the role of interoception in addiction and to develop new methods for studying how individuals with addiction process and perceive internal bodily sensations.

## Introduction

Interoception refers to the ability to perceive and understand the internal physiological state of the body, such as heart rate or hunger, that helps maintain homeostasis [1, 2]. It involves processing signals from multiple sources at neural, behavioural, and higher-order levels [2, 3] (see Table 1 for an overview). There has been increased interest in exploring the relationship between interoception and addictive behaviours in recent years, with many studies, articles, and opinions published on the topic [e.g. [4, 5••, 6–9, 10••]]. But how do interoceptive views of addiction link with older views on the role of impulsivity in addictive behaviours? In this article, I intend to examine the latest evidence on the role of interoception and impulsivity in addiction and their mutual influence on each other.

**Table 1:** An overview of the interoceptive dimensions referred to in this paper. The summary is based on [2, 3]

Dimension	Definition	Assessment methods
Neural representation	Central nervous system activity related to interoceptive processing, including the neural activation while attending to interoceptive sensations	Neuroimaging
Strength of afferent signals	The strength and nature of signals coming from the periphery conveying internal sensory information to the central nervous system	Peripheral physiological recordings (e.g. electrocardiogram, skin conductance)
Preconscious impact of afferent signals	The effect of afferent signals fluctuations on the processing of external stimuli and neural activity	Time-locking brief stimulus presentations to physiological events (e.g. cardiac contraction), manipulation of organ physiology
Interoceptive accuracy/sensitivity	The ability to precisely and correctly monitor changes in internal signals, such as heart rate; i.e. the correspondence between objectively measured physiological events and individuals’ reported experience of them	Behavioural tasks (e.g. heartbeat perception tasks, breathing load detection tasks)
Self-report/ Interoceptive sensibility	Subjective beliefs concerning individuals’ interoceptive sensations and experiences, self-reported assessment of bodily sensations in daily life	Questionnaire measures, confidence ratings on performance in behavioural tasks
Interoceptive insight	Metacognitive evaluation, i.e. the correspondence between subjective experience and objective performance, in other words: the extent to which subjective assessment tracks objective accuracy	The correspondence between accuracy during an interoceptive task, and performance confidence during the task
Interoceptive attention	The ability to direct attentional resources towards the source of internal body sensations	Neuroimaging, self-report, experience sampling methods
Attribution of interoceptive sensations	Interpretation of interoceptive cues and their causes	Self-report measures
Interoceptive abilities	A global term that reflects the ability to access, identify, understand, and respond appropriately to the patterns of internal signals (therefore it combines interoceptive accuracy, attention, sensibility, insight and attribution)	

## A Brief Outline of Neurocognitive Models of Addiction

The field of neurocognitive models of addiction has long recognized that addictive behaviours are related to an imbalance between two interacting, yet distinct, systems [e.g. [11–13]]. These dual models identify an “impulsive” system, driven by the amygdala-striatum (dopamine) pathway, that promotes automatic and habit-forming behaviours such as drug use, and a “reflective” system, dominated by the prefrontal cortex, responsible for decision-making, planning, and inhibiting actions. The models suggest that the root cause of addictive behaviours is an imbalance between these two systems, where the impulsive system becomes overactive due to repeated exposure to the drug or the reflective system becomes underactive due to frontal dysfunction caused by substance abuse.

The traditional view of addiction being controlled by two systems—impulsive and reflective—was challenged by a landmark study in neurological patients, which showed that smoking addiction was impacted by damage to the insular cortex [14••]. This led to the proposal of a triadic model, which considers the insular cortex as a key component in the integration of internal bodily states into conscious feelings and decision-making [15, 16]. The insula, particularly the anterior part, is known to play a crucial role in processing interoceptive cues—signals from the body that provide conscious access to subjective feelings [1, 17]. In drug users, these cues become strongly associated with the rewards of drugs, so damage to the insula may reduce the power of these conditioned stimuli to generate cravings [16, 18, 19]. Smokers with insula damage have been shown to have fewer urges to smoke, present milder withdrawal symptoms, and be less likely to need nicotine replacement therapy [20].

It is crucial to note that the way insular cortex processes and interprets interoceptive signals is influenced by the peripheral neural circuits that transmit information from the body to the central nervous system. The vagus nerve is one of the main pathways through which interoceptive signals reach the brain [21], and altering its signalling should affect drug cravings. Animal studies have supported this idea, showing that disrupting the peripheral interoceptive pathway through vagotomy (vagus nerve resection) in a high-alcohol-drinking line of rats prevented them from experiencing relapse-like behaviours, while other bodily functions remained unchanged [22•]. This suggests that interrupting the peripheral interoceptive pathways alters the way animals process and respond to interoceptive changes and decreases the motivational impact of alcohol, making it less likely for relapse to occur.

Taken together, over the years, the neurocognitive models of addiction showed a dramatic change in conceptualizing drug use and its causes. Specifically, they shifted from views that focused primarily on the dual system, in which compulsive behaviours result from impulses overpowering the reflective system, to the triadic system model, which also emphasizes the significance of the communication between the body and brain in creating cravings and withdrawal symptoms. The triadic model of addiction provides an elegant framework linking impulsivity, or the broader concept of impulse control, and interoception in addiction. While the association between impulsivity and addiction is well-established, the relationship between impulsivity and interoception has received little attention. The following discussion is going to explore the complex relationships between these three components in more detail.

## Impulsivity and Addiction

**Fig. 1:**
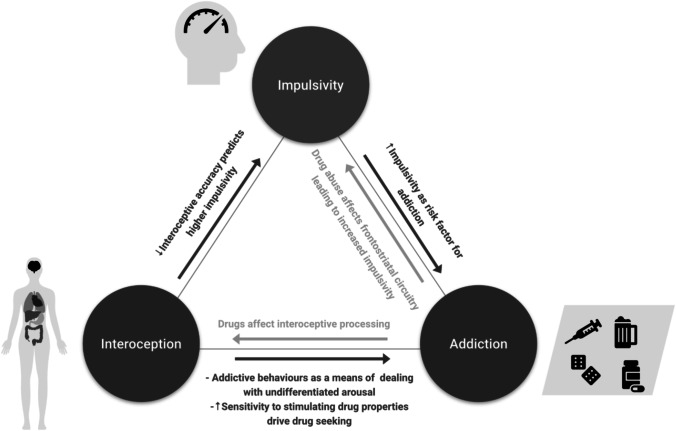
brief summary of evidence reviewed in this paper on the role of interoception and impulsivity in addiction and their mutual influence on each other.

Impulsivity is a complex and multi-dimensional phenomenon characterized by a lack of self-control and a tendency to act on immediate desires without considering consequences [23, 24]. It can be viewed as a fixed personality trait or a changeable, context-dependent, behaviour that can be divided into different subtypes: reflection impulsivity (a tendency to make rushed decisions in the context of uncertainty), temporal impulsivity (difficulty awaiting gratification), and motor impulsivity (difficulty waiting a turn to act or withholding a motor response) [for review, see [24]]. The connection between impulsivity and addiction has long been investigated as evidenced in many reviews and meta-analyses [e.g. [12, 25–29]]. These reviews show that impulsivity plays a significant role in addiction and can impact various stages of the addiction cycle. Impulsivity is considered to be both a risk factor for developing addictive behaviours [25–27] and a result of prolonged drug use that affects frontostriatal circuitry [12] (see Fig. 1 for an overview). It is important to note that the different facets of impulsivity may have varying relationships with addictive behaviours. For example, in the context of alcohol use, evidence suggests that increased impulsivity in terms of difficulty waiting for gratification, difficulty withholding an initiated response, and difficulty waiting for a turn to act serves as important risk factors for alcohol use [30]. Increased motor impulsivity also appears to be a result of alcohol use, but evidence is mixed for other forms of impulsivity [30].

## Interoception and Impulsivity

In contrast to the established relationship between impulsivity and addiction, the link between impulsivity and interoception has been investigated to a lesser extent. The interoceptive inference framework posits that the brain continually predicts the future state of the body and takes action to maintain homeostasis by reducing prediction errors [31, 32]. However, when these predictions are inaccurate, behaviour that goes against maintaining stability may result in self-control failures. Thus, if one is unable to detect, distinguish, and appropriately name feelings they are experiencing (i.e. show low interoceptive abilities), they may engage in impulsive actions as maladaptive means of dealing with this undifferentiated arousal [30]. Experimental work supports the link between interoception and impulsivity: higher accuracy in discriminating internal (heartbeats) from external signals (sequences of auditory stimuli) was found to predict lower trait impulsivity levels [33]. Therefore, poor interoception may predispose to impulsive behaviours (see Fig. 1 for an overview).

## Interoception, Impulsivity, and Addiction

If low interoceptive abilities may be a predisposing factor for engaging in impulsive behaviours, they may then also contribute to addictions (see Fig. 1 for an overview). In line with this notion, evidence suggests that individuals who are more accurate in predicting their interoceptive state are better at controlling cravings and have higher levels of trait self-control [34, 35•]. This indicates that people with more precise interoceptive predictive models have better self-regulation and can better manage their cravings. In contrast, heightened sensitivity to stimulating properties of a drug paired with poor inhibitory control jointly contributes to susceptibility to future excessive drug use. Specifically, poorer inhibitory control was linked to greater stimulation and fewer sedation experiences from alcohol as well as greater euphoria and arousal from amphetamine [36, 37]. Feelings of stimulation following alcohol were also negatively associated with activity in brain regions involved in inhibitory control (supplementary motor area, insula, and middle frontal gyrus) during successful response inhibition [36].

The perception of internal bodily sensations is frequently altered in individuals with addiction. For example, abstinent patients addicted to alcohol, heroin, nicotine, or synthetic cannabinoids showed lower interoceptive accuracy compared to control subjects [9, 38, 39], yet higher interoceptive sensitivity was reported in cocaine smokers [40]. Importantly, both, atypically low and high interoception can lead to behavioural and emotional difficulties. While low ability to detect changes in internal environment has been linked to poor adaptation to stressful situations, lower emotional resilience and psychopathology [41–43], atypically high interoceptive sensitivity, as well as low interoceptive insight, has been linked to panic and anxiety disorders [42, 44–46]. Therefore, those with poorer interoceptive skills may be less affected by strong cravings but instead may be using drugs as maladaptive ways of dealing with undifferentiated or misidentified bodily arousal [30, 47•]. In contrast, individuals with substance use disorder who show high interoceptive accuracy are likely to experience intense drug urges, and may also be at elevated risk of negative mood or stress-induced relapse [6]. Thus, both diminished as well as heightened interoceptive abilities may predispose to addictive behaviours.

Substance use can in turn interfere with the brain’s ability to accurately process and regulate interoceptive information [48] and produce changes in the brain’s reward system, resulting in decreased sensitivity to the body’s natural reward signals and altered sensitivity to the effects of drugs [49]. Indeed, changed neural processing within the interoceptive brain network of various, pleasant and aversive, interoceptive stimuli has been reported in individuals with substance use disorder and has been shown to vary depending on the stage of addiction [50–53]. Interoceptive deficits were also found to be positively correlated with addiction severity (31), implying that substance use may drive interoceptive deficits. This shift in interoception can then compel an individual to seek out drugs in order to experience pleasure and relief from negative internal states [54].

Interoceptive processing is at the heart of the use of substances to prevent or relieve negative emotional states resulting from abstinence or stressful environmental circumstances [55]. In turn, the psychophysiological effects drugs of abuse have on the body (feeling high) can lead to a feedback loop, where the pleasurable sensations associated with drug use affect interoceptive processing, making it even harder for individuals to control their drug-seeking behaviours [54]. Substance abuse further disrupts the balance between interoception and exteroception (the perception of external stimuli), leading to hyperfocus on external stimuli and a decreased ability to perceive internal bodily sensations (54). This can result in the inability to recognize the harmful effects of drug use, making it more challenging for individuals to quit. Repeated substance use (initially driven by pleasure) finally leads to the development of tolerance and the emergence of negative affect and consequently converts the use of drugs from an impulsive to a compulsive mode of action (negative reinforcement) [56].

Notably, the relationship between interoception, impulsivity, and addiction may not only apply to substance dependence but can also extend to behavioural addictions. Behavioural addictions, such as gambling, shopping, or internet addiction, activate the brain’s reward system in much the same way as substance use and are similarly related to impulsivity and disinhibition [57]. Current research on interoceptive processing in behavioural addictions is scarce and mixed (e.g. one study reported no differences in interoceptive abilities in a group with gambling disorder and matched controls [58], while another reported significantly lower interoceptive accuracy in problem gamblers [59]). Nevertheless, problem gamblers were found to exhibit attenuated physiological (skin conductance) responses to rewards compared to nonproblem gamblers [60, 61]. Such dampened afferent signalling to reward in problem gamblers may drive seeking further stimulation through gambling with larger amounts of money and for longer periods, presumably in order to experience the same excitement and level of arousal as nonproblem gamblers. Thus, interoceptive processing may also play a key role in the development and reinforcement of behavioural addictions but more research is needed to confirm that.

## Additional Reflections and Future Directions

Current research on interoception in relation to addiction primarily focuses on whether addiction is associated with alterations in interoceptive accuracy and sensibility (see Table 1 for definitions). Interoceptive accuracy is commonly assessed with heartbeat perception tasks.^1^ However, since participants are instructed to relax and focus on detecting internal sensations during these tasks, they are in a calm state and not likely to be disturbed out of their homeostatic balance. This limits the relevance of interoceptive processing during these tasks as interoception, by definition, is a sense that helps to maintain a homeostatic state. Arguably, as individuals are unlikely to be perturbed out of their homeostatic balance while performing these tasks, focusing on subtle internal bodily sensations may not be relevant in these situations. Yet, very few studies (in general, not only in the context of addiction) employ perturbations in the homeostatic state to study its effects on interoception. An important exception to this is a study by Smith and colleagues [10••] that used a breath hold to cause interoceptive disturbance. Compared to a control group, people diagnosed with depression, anxiety, or substance use disorder showed reduced sensitivity to changes in interoceptive signals, indicating that interoceptive problems in psychopathology are most noticeable during times of homeostatic disruption. This highlights the importance of studying interoception in non-homeostatic states.

Interoceptive sensibility reflects the subjective assessment of one’s interoceptive abilities. Such self-report measures require good self-insight in order to provide meaningful results. Yet, metacognitive impairments and decreased self-awareness are known hallmarks of addiction [66, 67]. Indeed, questionnaire measures indicate enhanced interoceptive sensibility in alcohol use disorder, which poorly corresponds with the performance outcomes in objective tasks, indicating low interoceptive insight [9]. This misalignment between objective interoceptive accuracy and subjective beliefs stands out as a crucial aspect of addiction. Interoceptive training targeting the discrepancy between subjective assessment of one’s abilities and interoceptive accuracy could be useful in bridging this metacognitive gap, as exemplified by promising results in autistic individuals [68•].

Recent research efforts mainly focused on interoceptive accuracy and sensibility, but gave less attention to the attribution of interoceptive stimuli. Nevertheless, it may be just as important, or even more so, to understand how a person with addiction views and responds to internal bodily signals, rather than just their ability to detect them. Being able to sense bodily sensations does not always mean that an individual pays attention to these signals or interprets them in a meaningful way that would lead to adaptive behavioural responses. For example, someone may accurately detect changes in arousal, but ignore them, or be poor at detecting bodily changes yet attach a lot of significance to their perceived sensations, which can lead to a negative interpretation of those feelings, as seen in panic disorder [44]. Interoceptive attributions can also affect cravings. Research showed that telling smokers they were getting nicotine in their cigarettes, as opposed to saying they received nicotine-free cigarettes, reduced their reported cravings, but only when they actually had nicotine [69]. These findings emphasize the substantial impact of beliefs on subjective cravings in smokers.

Importantly, bodily sensations can often be ambiguous, for instance, an increase in heart rate could be interpreted as resulting from exercise, heart problems, or excitement [44]. How the sensation is interpreted affects behaviour. Individuals with addictions may have a tendency to view ambiguous bodily sensations in a negative way and use substances or other unhealthy behaviours as coping mechanisms. Limited research has been done on this topic in the context of addiction, but one study during the COVID-19 lockdown found that people who had a negative outlook on the pandemic and struggled with mental resilience were more likely to drink alcohol to cope with stress [70]. It may be that this negative outlook also extends to bodily sensations in addictions. In fact, teenagers with substance use disorder have been shown to be hypersensitive to aversive interoceptive stimuli both at behavioural and neural levels [52], providing some support for this idea. Such negative interpretations may play a vital role as physical sensations related to anxiety or stress can lead to impulsive behaviour [24], possibly as a way to relieve discomfort. This highlights the importance of accurately interpreting bodily sensations in adaptive behaviours and the need to develop new methods to study this.

Another challenge is finding a reliable way to assess how individuals with addictions experience and distinguish different bodily sensations. The emBODY tool [71] may offer a solution. This tool allows assessment of bodily feelings by allowing participants to identify areas of the body in which (and with what intensity) they feel sensations, by creating individual maps of bodily feelings. Research using this tool has shown reliable emotion-specific patterns of sensations [71]. To my knowledge, the emBODY tool has only been used in a research context so far. However, this approach may help distinguish how different individuals experience emotions and drug-related symptoms, such as feeling high or withdrawal symptoms. Early results suggest that students with harmful alcohol use patterns experience more diffuse sensations for emotions and physiological states compared to low-risk drinkers [72]. Moreover, statistical classifiers distinguished feeling-specific activation maps less accurately for hazardous drinkers than low-risk drinkers, confirming that higher alcohol use is related to higher confusion of emotional and non-emotional bodily feelings. This confusion in bodily sensations may contribute to alcohol use as a way of dealing with undifferentiated changes in psychophysiological arousal during emotional states and maintaining emotional problems and alcohol (ab)use. In the future, this approach may be useful to study populations with addictions and also to track progress in addiction therapy settings.

## Novel Interventions Targeting Interoception and Impulsivity

Given the importance of both impulsivity and interoception in addictions, novel therapies should be targeting them. For example, mindfulness and meditation practices have been shown to increase interoceptive sensitivity and reduce impulsivity [73, 74]. This type of therapy is based on the principles of mindfulness meditation, which involves paying attention to the present moment without judgment. The goal of mindfulness-based therapy is to help individuals develop a greater awareness of their thoughts, feelings, and physical sensations, which can lead to increased control over their behaviours and a reduction in addictive tendencies. Thus, mindfulness-based interventions aim to help individuals to be more aware of their internal bodily sensations and, thus, regulate their behaviours accordingly. An alternative strategy could be to focus on improving interoceptive sensitivity through direct interoceptive training in people with substance use problems. To the best of my knowledge, this approach has not yet been evaluated in the context of addiction. However, a recent study showed that a 3-month cardiac interoceptive training program led to increased interoceptive sensitivity, improved alignment between objective and subjective measures of interoception (interoceptive insight), and reduced anxiety levels in autistic individuals [68•]. Improving the ability to read physical sensations and reducing anxiety levels, potentially by enhancing control over internal stimuli, could also be helpful for individuals with addiction.

Technological advancements have additionally opened up new possibilities for treating addiction through direct targeting of the insular activity with non-invasive methods like transcranial magnetic stimulation (TMS) or transcranial direct current stimulation (tDCS). So far, the results are encouraging, but further research is necessary to establish their full potential in treating addiction [75]. This includes refining the therapeutic methods (frequency, dosage, and location of stimulation) and exploring the possibility of personalized treatment approaches. Another interesting method for targeting insula activity has been suggested that does not involve specialized equipment: This approach involves intense physical exercise, which has been shown to cause changes in insular activity and potentially decrease the heightened internal response to stimuli related to drug use [76].

Finally, because interoceptive pathways play a crucial role in transmitting signals from the body to the brain and are significant in addiction, the peripheral interoception route could provide a new option for treating addiction. Translational research suggests that invasive procedures like vagotomy (surgical removal of the vagus nerve; see above for details) can be effective [22•]. However, a non-invasive method for manipulating vagal nerve activity, known as transcutaneous vagal nerve stimulation (tVNS), is also promising. Studies in humans have shown that tVNS can significantly reduce withdrawal symptoms, pain, and distress levels, and decrease the risk of relapse in individuals with opioid addiction [77]. Furthermore, tVNS has been linked to improved cognitive control processes, which are important for regaining control over drug use (reviewed in [78]). Given its ability to reduce the behavioural and physiological effects of withdrawal and improve cognitive control, tVNS has great potential as a readily available and easily implementable adjunctive treatment for addiction, as it can be used like an earpiece and requires minimal medical supervision outside of clinical settings.

## Conclusions

Figure 1 provides a brief summary of evidence on the role of interoception and impulsivity in addiction and their mutual influence on each other. In conclusion, interoception plays an important role in the development and maintenance of addictive behaviours. Altered interoceptive processing across different levels, neural, behavioural, and higher-order, may predispose to drug use via its influence on impulsivity and by promoting maladaptive coping strategies to deal with undifferentiated arousal. In turn, disruptions to interoception due to substance abuse can lead to difficulty regulating drug-seeking and impulsive behaviours. Understanding the role of interoception in addiction can help clinicians and researchers develop more effective treatments for addiction and fine-tune existing ones. Interventions that target interoception, such as mindfulness and meditation, as well as those that target insular activity or interoceptive peripheral pathways directly show promise in improving interoceptive sensitivity, reducing impulsivity, and helping individuals overcome addiction. Future research should put more emphasis on the perception, appraisal, and interpretation of interoceptive stimuli in addiction as they affect the individual’s behaviour and their use of substances or other unhealthy coping mechanisms. Despite a growing interest in interoception in addiction research, further research is needed to fully understand its role in addiction, particularly behavioural addictions, and to develop new methods to study the way individuals interpret various internal sensations in addiction.

## Footnotes

1 Currently, the validity of these tasks is under heated debate and new approaches are being developed. Discussion of these issues is beyond the scope of this review, but I encourage interested readers to review the following papers on the topic: [62–65].
